# Cytomegalovirus Infection Screening in Pregnant Women from Northwest Romania: Results of a 15-Year Follow-Up Program

**DOI:** 10.3390/microorganisms13071513

**Published:** 2025-06-28

**Authors:** Monica Muntean, Violeta Tincuța Briciu, Angela Monica Ionică, Melinda Horvat, Mihaela Lupse, Amanda Radulescu

**Affiliations:** 1 Infectious Diseases and Epidemiology Department, “Iuliu Hațieganu ” University of Medicine and Pharmacy, 400012 Cluj-Napoca, Romania; monica.muntean@umfcluj.ro (M.M.); melinda.horvat@umfcluj.ro (M.H.); mihaela.lupse@yahoo.com (M.L.); aradulescu@umfcluj.ro (A.R.); 2 Clinical Hospital of Infectious Diseases, 400348 Cluj-Napoca, Romania

**Keywords:** Cytomegalovirus, primary infection, prenatal screening, anti-cytomegalovirus antibodies

## Abstract

Pregnancy-related cytomegalovirus (CMV) infection may have negative consequences on the developing fetus and child. In Romania, screening for CMV infection is included in the first prenatal evaluation. We aimed to evaluate the seroprevalence rates in pregnant women who underwent screening over 15 years (May 2008–February 2023). We evaluated 28,012 pregnant women, from whom 23,970 (85.57%) had an index CMV screening, and 4082 had at least two presentations during the same or consecutive pregnancies. A total of 32,290 paired anti-CMV IgM/IgG serological tests were performed. Passed infection with IgG positivity represented 90.15% (29,110) of all tests, corresponding to 28,649 women (88.72%). The seroprevalence increased with women’s age, was more frequently associated with rural residence, and decreased in time intervals. A total of 2322 women (9.69%) displaying an IgM/IgG negative pattern were at risk of acquiring the infection during pregnancy. Only 144 out of 2342 (6.14%) women at risk presented twice during the same pregnancy, of which 12 women (0.51%) displayed a pattern of primary infection. Our population from Northwest Romania shows a high rate of immunization against CMV infection and a low risk of primary infection. We found a low adherence to retesting in cases of probable primary CMV infections, which highlights the need for infection mitigation by hygiene measures and improvement of the existing protocols.

## 1. Introduction

Congenital CMV (cCMV) infection is the leading congenital viral infection worldwide, with an estimated incidence between 0.2% and 1% of all live births [1]. Cytomegalovirus (CMV) infection affects 0.4–5% of all live newborns depending on the country, and up to 10% may present symptoms at birth [2].

The prevalence of live births with cCMV is three times higher in countries of low to medium socio-economic status than in developed countries [3,4]. Congenital CMV infection is the leading cause of non-genetic sensorineural hearing loss and one of the leading causes of neurodevelopmental delay with mental or motor impairment and microcephaly [1,5,6,7,8]. It accounts for up to 10% of all cerebral palsy cases [9] and 8–21% of neurosensory deafness cases at birth [10,11,12,13,14].

CMV is a double-stranded DNA herpesvirus that is endemic worldwide. Like all herpesviruses, CMV has biological properties of latency and reactivation, and, therefore, is a lifelong infection [15,16]. There are three types of infection: primary, when the mother has previously tested negative for CMV (IgG and IgM) and seroconversion occurs during pregnancy; reactivation of the latent virus; and reinfection with a new strain in patients with previous contact, the last two of which are considered non-primary infection [3,17,18,19,20]. Five distinct genotypes circulate throughout the world and previous CMV infection does not prevent reinfection with another strain. Both primary infection and non-primary infection (reactivation or reinfection) during pregnancy may cause intrauterine transmission, although the risk is greater for primary infection (30–40%) than for non-primary infection (0.2–3%) [2,3,21,22]. The risk of transmission increases with gestational age (with a similar pattern as for other congenital infections like toxoplasmosis or rubella), although the risk of permanent postnatal sequelae is mainly driven by periconceptional and first trimester infections [23].

The most common way of transmission of infection is through direct contact with a person excreting the virus in the saliva, urine, or body fluids. The greatest risk factor for transmission in women of reproductive age is exposure to the contaminated saliva and urine of young children, with a risk up to 10 times higher than in other unexposed groups [3,24].

Despite the extensive knowledge regarding the epidemiology and pathogenesis of CMV infections in pregnant women, the risk of infection is poorly understood by the general population compared to other, less common conditions such as Down syndrome or spina bifida [3,25].

This shows that health professionals and governments have not run campaigns to prevent and raise public awareness about this infection. Since no vaccination is currently available and the present treatment is not yet broadly approved by the scientific community, this strategy would be the most effective way to prevent cCMV infection [3,17,26,27].

CMV screening can be performed by testing specific antibodies (IgG, IgM, and IgG avidity) and/or by detecting cytomegalovirus DNA in body fluids (amniotic fluid, blood, urine, and saliva) [18,28,29]. Only IgM positivity is not considered a reliable marker of primary infection since it may persist for many months, may be detected in non-primary infections, may exhibit cross-reactivity with other viral infections, and polyclonal immune stimulation [26].

We defined the type of infection based on the serological profiles suggested by Sanchez-Duran and Maltezou [2,30], as follows:

Primary infection: any of the following:

- Seroconversion during pregnancy (negative/positive IgM and negative IgG in the first sample and positive IgM and IgG in the second at more than 2–3 weeks);
- IgG and IgM positive with low or intermediate IgG avidity;

Passed infection (seroprevalence): any of the following:

- IgG-positive and IgM-negative;
- IgG- and IgM-positive with high IgG avidity index.

False positive tests: IgM-positive and IgG-negative in paired tests at least two weeks apart.

Guidelines and practices regarding CMV serological screening in pregnancy are heterogeneous. Although most of the North American and European guidelines do not recommend routine screening for CMV infection during pregnancy, in some areas of France, Italy, Belgium, Israel, Spain, Italy, Germany, Austria, Portugal, and the Netherlands, routine CMV serological screening is provided [1,2,5,25]. In the absence of clinical signs of infection, the serological diagnosis of this infection is challenging, which brings potential harm in the form of the unnecessary recommendation of termination of pregnancy (TOP), which is a major roadblock for implementing universal screening [6,26,31,32].

A systematic review of the literature carried out by Xie et al. in 2023 regarding the existence of guidelines and consensus for CMV screening during pregnancy found that none of the 13 included studies suggested universal screening. Eight guidelines and two consensuses were against universal testing in this population. Five guidelines recommend targeted screening only for patients at high risk of infection, i.e., pregnant women who have children up to 3 years of age or who work in daycare centers [21]. Moreover, the guidelines differ on how testing is performed, mentioning two types of approaches: the first using IgG, IgM, and IgG avidity testing, and the second using only specific IgG testing [33,34,35,36,37].

In Central-Eastern Europe, recent studies have reported varying seroprevalence rates, ranging from a low of 57.3% in Poland [38] to a high of 93.5–95.7% in Romania [39,40].

In Romania, TORCH screening (toxoplasmosis, syphilis, HIV, CMV, herpes, rubella, hepatitis B and C) is included in the first antenatal visit, as recommended by the Health Ministry and reimbursed by the National Health Insurance System, and there is no further official recommendation for a retesting protocol [41,42]. Mocanu et al. conducted a TORCH seroprevalence study in Western Romania, covering two periods: 2008–2010 and 2015–2018. The study involved a total of 6961 pregnant women. The seroprevalence of IgG antibodies against *Toxoplasma gondii* decreased from 43.7% to 38.8%. Similarly, the prevalence of IgG antibodies against CMV declined from 94.7% to 91.1%, while those against the rubella virus fell from 94.1% to 91.5% [43]. Briciu et al. reported a declining trend in IgG anti-toxoplasma antibodies in a significant study conducted in Northwest Romania that involved 27,169 pregnant women. The prevalence decreased from 30.04% to 27.39% over five-year intervals during the study period from 2008 to 2023 [41].

In Western Europe, the seroprevalence ranges from 45.6% in France [44] to 67.1% in Norway [45].

In terms of prevention, there is no licensed vaccine for CMV. Two randomized controlled trials showed only modest (40–50%) efficacy in reducing the risk of CMV in young women [26].

Studies on the treatment of pregnant women with seroconversion in the periconceptional period and in the first trimester of pregnancy with high doses of valacyclovir (8 g/day) have been introduced in the active research [28,46,47].

A complementary strategy to reduce the risk of CMV infection is behavior changes to minimize direct contact with saliva and urine from other people, especially from young children. Hygiene-based measures are considered of the utmost importance, including sharing with care drinks or food already tasted by other persons, avoiding sharing utensils, kissing young children on the forehead instead of directly on the lips, and washing one’s hands after contact with urine or saliva [31,48,49,50,51].

## 2. Materials and Methods

### 2.1. Study Design and Setting

We conducted a retrospective analysis of all consecutively examined female patients who underwent prenatal screening of anti-CMV antibodies in The Clinical Hospital of Infectious Diseases, a large Tertiary Center located in Cluj County, North-Western Romania.

The Ethics Committee of the Clinical Hospital of Infectious Diseases Cluj-Napoca approved our study through decision 3279/27.02.2025.

The serology testing for TORCH (*Toxoplasma gondii*, rubella, cytomegalovirus, herpes simplex virus 1, 2) was performed in the hospital ambulatory starting in 2008. The national protocol recommends prenatal CMV testing, preferably in the first trimester of pregnancy. Even if not mentioned in the national protocol, retesting is recommended in seronegative women and, in the case of IgM/IgG CMV positive results, should be completed with avidity tests. Counselling regarding keeping a CMV seronegative status during pregnancy and possible further investigations is also provided.

### 2.2. Participants

We retrospectively included all pregnant women who presented for prenatal screening between 14 May 2008 and 31 December 2023 by accessing the hospital electronic database. Their identification was based on the TORCH protocol for pregnant women, coded as Z36.9 (International Statistical Classification of Diseases and Related Health Problems 10th Revision).

### 2.3. Variables

We collected data on age, demographic (urban/rural, county), date of testing, IgM, IgG anti-CMV antibodies, and avidity IgG test results when performed. Consecutive testing during the same pregnancy and in subsequent pregnancies was done in compliant participants.

### 2.4. Laboratory Assays

The anti-CMV (IgM and IgG) antibody screening was performed using the fully automated Alinity, a 2-step chemiluminescent microparticle immunoassay (CMIA), Abbott Ireland Diagnostics Division, Sligo. If the IgM result was positive or borderline, another qualitative test was performed using the automated analyzer VIDAS, a two-step enzyme immunoassay sandwich method with a final fluorescent detection (ELFA), BIOMÉRIEUX, Craponne, France. For IgM positive/borderline and IgG positive results, the IgG avidity test was recommended, and was conducted using the automated VIDAS CMV IgG Avidity II, ELFA technique (Enzyme Linked Fluorescent Assay), BIOMERIEUX, Craponne, France. The avidity index values were classified as low (<0.40), borderline (0.40 ≤ index < 0.65), or high (≥0.65).

### 2.5. Statistical Analysis

The statistical analysis was performed using EpiInfo™ 7.2 software (CDC, Atlanta, GA, USA). Global and group-specific calculations were conducted to determine the prevalence and 95% CI. The chi-squared test was used to evaluate the prevalence differences, and a *p*-value of less than 0.05 was considered significant. The first positive test or the first test given to patients who consistently scored negatively was considered for further calculations for women who presented more than once. A freely accessible web tool was used to create evolution diagrams in the case of many presentations [52].

## 3. Results

During the study period, 28,012 pregnant women presented for prenatal screening. The majority (23,970 women; 85.57%) had an index CMV testing. A total of 4082 women had more than one presentation as follows: at least two times during a single pregnancy (144), once during each of the recorded consecutive pregnancies (3897), or repeatedly in at least one of the consecutive pregnancies (40). The demographic characteristics are presented in Table 1.

**Figure 1:**
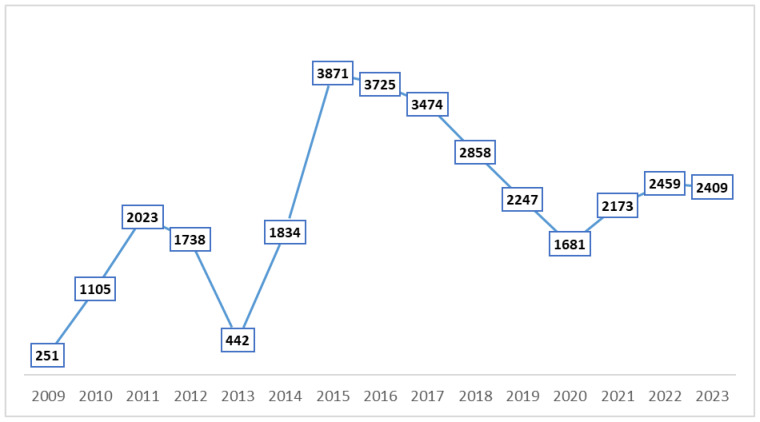
The annual distribution of anti-CMV IgM and IgG antibody tests.

A total of 32,290 anti-CMV IgM and IgG antibody serological tests were performed. The annual distribution of the tests is shown in Figure 1.

The majority (22,575; 70%) were pregnancies carried out in the 25–34.9 age group. A total of 29,110 tests (90.15%) were positive for IgG antibodies (Table 2). The proportion of IgG-positive tests slightly increased with age, and the overall differences among age groups were statistically significant (χ^2^ = 26.29; df. = 8; *p* = 0.0009). The seroprevalence was significantly higher (χ^2^ = 38.47; df. = 1; *p* < 0.0001) in women with a rural residence (7836; 91.78%; 95% CI: 91.18–92.34), as compared to those living in urban areas (17,403; 89.37%; 95% CI: 88.92–89.79).

Overall, 272 tests (0.84%) were positive/borderline for IgM. There were no statistically significant differences between age groups (χ^2^ = 19.41; df. = 8; *p* = 0.25), but a significant difference was found between rural (89; 1.04%; 95% CI: 0.66–0.91) and urban (151; 0.78%; 95% CI: 0.85–1.28) residents (χ^2^ = 4.67; df. = 1; *p* = 0.03).

The seroprevalence of IgG antibodies significantly decreased during the considered time periods (Table 3).

In total, 3151 tests were negative for both IgM and IgG antibodies, while 272 (0.84%) were positive for both types of antibodies and 189 (0.59%) tests were IgM borderline and IgG positive (Table 4).

### 3.1. Index CMV Testing in Pregnant Women

Out of the 23,970 women who showed up for screening once, 21,304 (88.88%) displayed a pattern of passed infection (IgM negative/IgG positive) and 2322 women (9.69%) were IgM and IgG negative, which means that they had a potential risk of primary infection later in pregnancy.

**Figure 2:**
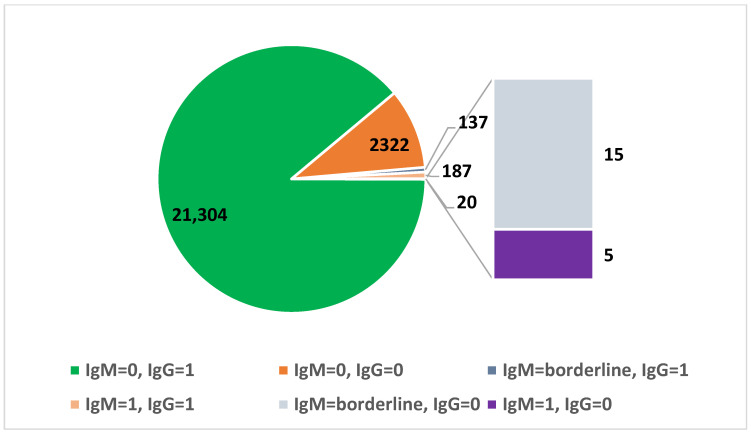
IgM and IgG serologies in pregnant women who had an index CMV screening test at our medical unit.

A pattern of potential CMV primary infection was found in 344 women (1.44%) who displayed an IgM positive/borderline and IgG negative profile or IgM positive/borderline and IgG positive tests (Table 5 and Figure 2).

### 3.2. Serological Profiles During the Same Pregnancy

**Figure 3:**
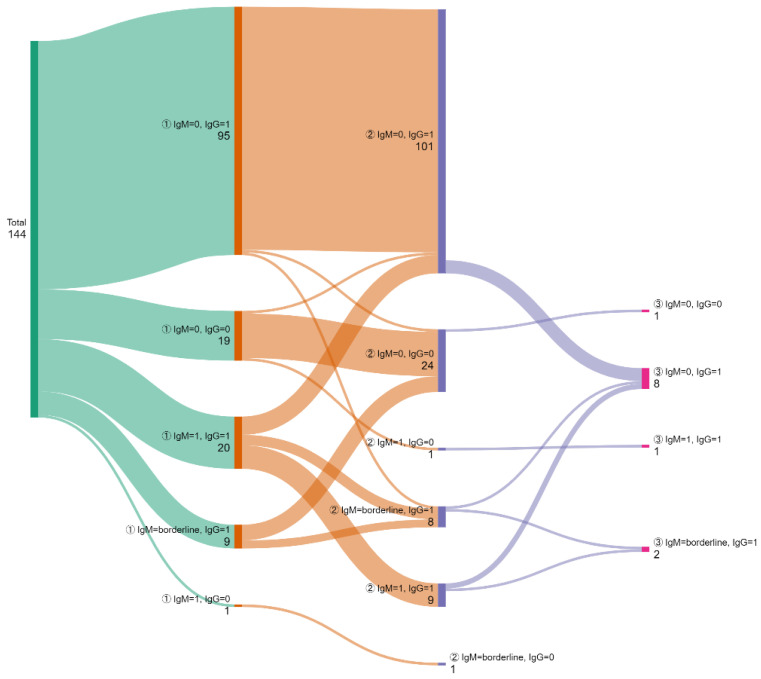
The dynamics of test results in women with multiple presentations during the same pregnancy. The number of patients is presented next to each serological profile. 0 = negative; 1 = positive. The circled numbers represent the number of the screening test (from the first up to the third).

Overall, only 144 women presented twice during the same pregnancy, out of which 12 showed up three times. Their serology dynamics are presented in Figure 3. Most tested women (106; 73.6%) had a non-primary (passed) CMV infection defined by an IgM negative/IgG positive profile. Three of the profiles that were defined as IgM negative/borderline and IgG positive in paired tests have been classified as previously infected. At both presentations, 17 women displaying an IgM/IgG negative profile were at potential risk of primary CMV infection.

Between the first and second presentations, and according to the proposed classification [18,28], there were two confirmed primary CMV infections that showed an IgM positive/IgG negative profile with seroconversion to IgM negative/IgG positive (one case) and an IgM/IgG negative profile with seroconversion to IgM positive/IgG negative (one case). Presumed primary CMV infections suggested by an IgM positive/borderline and IgG positive profile at the second presentation were found in 16 women, of whom 10 displayed a profile of primary CMV infection based on a low or intermediate avidity test.

Among the 12 women tested three times, one was found to have a primary CMV infection, based on seroconversion from an IgM/IgG negative profile to an IgM/IgG positive profile. Three women showed the same profile at all evaluations, namely IgM positive/IgG positive, being considered previously infected, while seven were classified as having prior CMV infection based on IgM negative/IgG positive at all presentations. One other woman who displayed an IgM/IgG negative profile had a potential risk of primary infection.

### 3.3. Serological Profiles During Consecutive Pregnancies

**Figure 4:**
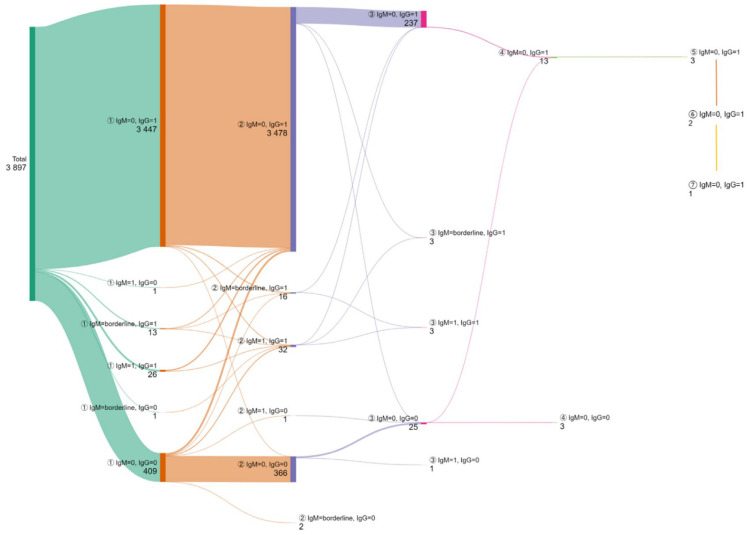
The dynamics of test results in women with multiple pregnancies. The number of patients is presented next to each serological profile. 0 = negative; 1 = positive. The circled numbers represent the number of the pregnancy (from the first up to the seventh).

A total of 3897 women were evaluated for two consecutive pregnancies, from which 269 (6.9%) for three pregnancies and 32 were for more than three pregnancies (Figure 4).

The majority (3447; 88.45%) were confirmed to have a non-primary infection, being IgG positive/IgM negative during two consecutive pregnancies. A total of 365 (9.36%) women remained uninfected during the two consecutive pregnancies, displaying an IgM/IgG negative profile.

Between the first and second pregnancies, 0.44% of women (17/3897) seroconverted to IgG antibodies, and 0.84% (33/3897) maintained the same profile (IgM positive/IgG positive) as in their first pregnancy. No seroconversion to IgG was found in any of the 269 women who were tested in more than two pregnancies, and IgG became undetectable in an additional woman. In eight women, a persistent IgM positive/IgG negative profile was found, and of these eight women, six had two pregnancies and the others had three.

A total of 116 avidity tests that displayed IgM/IgG positivity were performed in 81 women during the same pregnancy, of which 71 had high values, six had borderline values, and four had a low index value.

## 4. Discussion

Our study provides data on a substantial number of pregnant women tested for CMV with reliable serological tests that were performed in a tertiary infectious disease hospital during a 15-year interval. We undertook the largest study on CMV infection in Romania to date, evaluating 28,012 women and performing 32,290 IgM/IgG CMV-antibody tests. We present data that partially cover the Transylvania region, with the largest representation being of Cluj County (85.28%), with both urban (69.52%) and rural residences. The mean age of the women at CMV testing was 29.44 ± 4.96 years, and the corresponding groups of age represented over two-thirds of the participants, like other studies from Romania [39,40,53].

According to our statistics, the percentage of women who tested positive for IgG was high (90.15%) and decreased minimally but statistically significantly (91% to 88.99%) throughout the duration of the study. Based on a meta-analysis, Zuhair et al. estimated the global prevalence of this infection in women of childbearing age to be 86% (95% UI: 83–89), with the highest seroprevalence being in the Eastern Mediterranean World Health Organization region (90% [95% UI: 85–94]) and the lowest being in the WHO European region (66% [95% UI: 56–74]) and in the UK, Germany, Canada, Ireland, the Netherlands, Belgium, and France [17]. The region of the Americas aligns with the global values (79%; 95% CI, 69–87%), with the highest CMV prevalences being in Brazil (98.1%; 95% CI, 97.4–98.7%) [17,54] and Chile (92–95%) [55].

Taking Germany as an example of a country with a low IgG CMV prevalence, Enders et al. found an overall CMV IgG seroprevalence of 42.3% in research involving 40,324 pregnant women aged from 15 to 50 years from 1996 to 2010. Over the study period of 15 years, the seroprevalence in pregnant women significantly declined from 44.3% in the first interval period (1996–2000) to 42.8% (2001–2005) and to 40.9% (2006–2010) [32]. Another study conducted in Bavaria, Germany, found that the rate of CMV screening during pregnancy was 40.3%, and detected IgG seropositivity in 35.5% (143/403) of women [56].

Even though our research showed a statistically significant decrease in IgG CMV seroprevalence, the rate remained remarkably high at 88.99% throughout the last five study years. Moreover, a considerable number of women (3897) were evaluated during two consecutive pregnancies, and the majority (88.45%) were confirmed to have non-primary infection. Our results are in line with other studies from different regions in Romania. A study from Southwest Romania that evaluated CMV infection in two periods (2013–2016 and 2019–2022), including 3404 pregnant women, found an increasing trend of IgG antibodies from 93.68% to 94.96% [39]. In Western Romania, Mocanu et al. evaluated 6961 pregnant women and discovered that the IgG anti-CMV prevalence decreased from 94.7% to 91.1% in two successive intervals, 2008–2010 and 2015–2018 [53].

Similar to other studies conducted in Romania, we observed a significantly higher seroprevalence in women with a rural residence as compared to those living in urban areas [39,40,53].

This finding may be related to a lower educational and economic level in rural areas, as was found in studies all over the world [32,57]. This is not always the case in the WHO European region, as Greye et al. found that, between 2014 and 2018, the prevalence of IgG in rural Germany was 45% [58]. There may be a small upward or downward trend in IgG seroprevalence, but Romania may fit into the high-prevalence WHO Eastern Mediterranean region. The high rate of passed infection is not reassuring because of the rate of non-primary CMV infections (reactivations and reinfections), for which there is a positive correlation between birth cCMV prevalence and population seroprevalence [59]. In the most recent meta-analysis, covering studies from 36 countries (five continents), Ssentongo et al. estimated the birth prevalence of cCMV as being three-fold higher in low- and middle-income countries than in high-income countries, and found that a higher maternal CMV seroprevalence was associated with higher cCMV rates (OR, 1.22; 95% CI, 1.05–1.40). The conclusion was that non-primary CMV infection plays a significant role in the burden of cCMV [4]. This seeming paradox may be explained by the risk of reactivation during pregnancy, which, even if infrequent, might be substantial at the population level, and risky behavior having a higher risk of reinfection in a highly seropositive population [4,22].

The percentage of IgM positivity/borderline was very low, 0.87%, was significantly higher in rural residents, and there was no difference between age groups. It is generally accepted that IgM positivity is not a trustworthy marker for primary infection because of long-term persistence and a high rate of false positivity. IgM/IgG positivity with a low avidity IgG index is also of limited value if the titer of IgG is low and the interpretation of the intermediate avidity index is also challenging [31,48,60].

A total of 2322 (9.69%) women in our study were at potential risk of primary CMV infection during pregnancy as no CMV-IgG antibodies were detected. Even though the risk of fetal anomalies after primary infection in the second and third trimesters of pregnancy is less than 1%, all women were counselled by an infectious disease specialist about risk mitigation through hygiene-based measures.

All women (344; 1.44%) who had a profile of potential primary infection, meaning IgM positive/borderline and IgG negative or IgM positive/borderline and IgG positive, were asked to undergo another test, preferably in a two-week interval. Adherence to retesting during the same pregnancy was overall very low, and somehow surprisingly, women with previous primary infections were more compliant to retesting in the same and consecutive pregnancies. The most probable explanation is poor compliance with the screening program and, conversely, for certain CMV-immunized women, over-vigilance, as reinfections and reactivations cannot be properly evaluated by serological testing in immunocompetent women [4,6,57].

In a semi-systematic review including 13 papers (screened from 764 studies), Bartnik et al. found that the general awareness and knowledge about CMV infection among pregnant women from developed countries was low (<40%), except for two studies from Italy and France showing that ~60% of respondents had knowledge regarding CMV infection. However, this may have been an overestimation because the correct answers regarding primary prevention measures were modest. Three studies showed that educational interventions proved to have a positive impact but without an analysis of their effectiveness [61]. Greye et al. evaluated the awareness of cCMV in German maternity departments with questionnaires addressed to 1233 women, of which 48.5% reported any knowledge about the risk of CMV infection during pregnancy, and only one third undertook serological testing. In contrast, better knowledge was found about other infections, such as toxoplasmosis (>93%) and listeriosis (>58%). Moreover, any education regarding CMV infection was reported only by 38% of respondents as compared to education about toxoplasmosis (>88%), listeriosis (>50%), and chlamydia (>64%) [62].

Among the few women (144) who were tested twice during the same pregnancy, the majority were either previously infected or not infected with a low but persistent risk of having a CMV-infected child due to a higher rate of transmission later in pregnancy. As was detailed in Figure 3, two women had primary infections based on the seroconversion profile, and 10 additional women had an IgM/IgG positive profile with a low or intermediate avidity index. Considering the risk of cCMV as a total of 12 cases in 144 pregnancies, if all women with a potential risk of primary infection had been assessed twice, the presumptive cumulative rate of seroconversion might have been 11.27% (264 out of 2342).

Mussi et al. assessed the cCMV infection in a highly IgG seropositive population from Brazil (98.1%; 95% CI 97.4–98.7%). They followed 1952 women who were evaluated at least twice, with the additional test collected at ≥14 weeks after the initial specimen was taken. The cumulative rate of seroconversion in seronegative women (5/36) was 13.9% (95% CI, 4.8–30.6%), which is comparable with our presumptive cumulative rate [54]. Congenital CMV infection was evaluated in 1721 neonates by the molecular testing of saliva (in the first week of age) and confirmed by urine test within the first 3 weeks of age. Congenital CMV infection occurred with a rate of 1 in 36 infants (2.8%; 95% CI, 0.5–14.2%) born to initially seronegative mothers and in 8 of 1685 (0.5%; 95% CI, 0.2–1.0%) infants born to seropositive mothers.

Consistent with the above study and worldwide statistics, most affected infants are born to mothers with pre-existing immunity, although the risk is lower and less frequent, while those born to mothers with primary infections possess a much higher risk of severe anomalies.

Therefore, serology seems to be more of a presumption than an accurate risk assessment, and congenital infections are likely to represent a mixture of primary and recurrent infections [2,4,22,29,54,59,63]; thus, the lack of guidelines and consensus [21,26,31,60].

Among the few women who were assessed for two or three consecutive pregnancies, CMV serology cannot be properly interpreted in those who displayed either prolonged IgM/IgG positivity or IgM positive/IgG negative profiles.

The early prenatal testing of whole-genome cell-free DNA (cfDNA) may confirm primary infection and ensure, in due time, treatment with valacyclovir, even though the time window is estimated to be as short as five weeks [1,18,21,60,64]. Faas et al. performed a large study on 204,818 women in early pregnancy who underwent non-invasive prenatal testing, fetal aneuploidy screening, and whole-genome cell-free DNA (cfDNA) sequencing stratified by viral load and confirmed by hCMV-qPCR, which may ensure the identification of women with active CMV infections and who are eligible for therapy. This strategy with one single blood test drawn in the first trimester of pregnancy might bring certainty for cCMV being added to aneuploidy screening, which has already been implemented in many countries and can be performed on a large-scale basis [29,65].

The key components of cCMV mitigation are general awareness and hygiene-based interventions, which emphasize the need to minimize contact with the urine and saliva of young children who may be shedding CMV [18,21,26,31,64].

Even though cCMV infection has serious consequences and is quite common in absolute numbers, pregnant women generally have little or no knowledge of this disease, and the doctors and nurses who care for these patients are not in the habit of recommending primary prevention. All pregnant women and women who intend to become pregnant need to be informed about the child risk and about ways to improve their hygiene to reduce transmission.

Considering the new diagnostic approach based on molecular testing in addition to serology, treatment with valacyclovir in documented infections may enhance maternal and child healthcare [26,28,47].

For now, we should focus on better primary prevention measures such as practicing hygiene and conduct the necessary groundwork to figure out whether the benefits of routine serologic screening and treatment outweigh the human and economic costs.

Along with other European countries, we promote the ongoing protocol of screening with additional molecular testing offered together with aneuploidy testing early in pregnancy.

## 5. Strengths and Limitations

The strengths of our study are represented by its sample size, which provides information on the largest number of pregnant women in Romania who have been screened for CMV infection in a cohort study over a 15-year interval. Along with other Romanian studies, our study presents new data on CMV seroprevalence in Northwest Romania.

The primary limitation of the study’s retrospective design is the lack of information regarding the week of pregnancy at the initial test. Although our national protocol recommends testing in the first 8–10 weeks of pregnancy, late presentation is not excluded. In women with a suggestive profile for primary CMV infection, we were not able to assess the clinical data of neonates and congenital infection through CMV detection in amniotic fluid, or urine in the first 2 weeks of age.

## 6. Conclusions

The seroprevalence of CMV infection is still high in Northwest Romania, and the decreasing trend is in line with the European profile. Low adherence to retesting limits the interpretation of serological evaluation, a focus that has not been addressed in other seroprevalence studies conducted in Romania. Our report provides insights into CMV follow-up assessment in a highly seropositive maternal population and emphasizes the necessity of developing intervention strategies for the prevention of maternal and congenital CMV infections that are culturally and economically proper.

## Figures and Tables

**Table 1:** Demographic characteristics of the screened population.

Age	Mean (min-max) ± SDEV	29.44 (12–54) ± 4.96
	Median	29
County	Cluj County	23,889 (85.28%; 95% CI: 84.86–85.69)
	Other counties	4123 (14.72%; 95% CI: 14.31–15.14)
Residence	Urban	19,474 (69.52%; 95% CI: 68.98–70.06)
	Rural	8538 (30.48%; 95% CI: 29.94–31.02)

**Table 2:** Test results according to age group.

Age Group	*n*	%	IgM			IgG		
			+	%	95% CI	+	%	95% CI
[12–14.9]	14	0.04	0	0.00	0.00–33.67	14	100.00	76.84–100.00
[15–19.9]	587	1.82	7	1.19	0.58–2.44	540	91.99	89.51–93.93
[20–24.9]	3766	11.66	36	0.96	0.69–1.32	3399	90.25	89.27–91.16
[25–29.9]	11,816	36.59	98	0.83	0.68–1.01	10,558	89.35	88.78–89.9
[30–34.9]	10,759	33.32	102	0.95	0.78–1.15	9701	90.17	89.59–90.71
[35–39.9]	4499	13.93	36	0.80	0.58–1.11	4115	91.46	90.61–92.25
[40–44.9]	812	2.51	3	0.37	0.13–1.08	747	92.00	89.92–93.67
[45–49.9]	35	0.11	0	0.00	0.00–10.00	34	97.14	85.08–99.93
[50+]	2	0.01	0	0.00	0.00–84.19	2	100.00	15.81–100.00
TOTAL	32,290		282	0.87	0.78–0.98	29,110	90.15	89.82–90.47

**Table 3:** Anti-CMV IgG seropositivity expressed in time periods.

**Period**	**Positive Tests**				**Positive Women**			
	***n***	**%**	**95% CI**	χ^2^ = 17.8 df. = 2 *p* = 0.0001	***n***	**%**	**95% CI**	χ^2^ = 19.48 df. = 2 *p* = 0.0001
I (2009–2013)	5063	91.08	90.30–91.80		4846	91.00	90.21–91.74	
II (2014–2018)	14,260	90.47	90.00–90.92		12,408	90.48	89.97–90.96	
III (2019–2023)	9787	89.22	88.63–89.79		7985	88.99	88.32–89.62	

**Table 4:** Crosstabulation of IgM and IgG screening test results.

		IgG					
		Negative			Positive		
		*n*	%	95% CI	*n*	%	95% CI
IgM	negative	3151	9.76	9.44–10.09	28,649	88.72	88.37–89.07
	borderline	19	0.06	0.04–0.09	189	0.59	0.51–0.67
	positive	10	0.03	0.02–0.06	272	0.84	0.75–0.95
Total		3180	9.85	9.53–10.18	29,110	90.15	89.82–90.47

**Table 5:** Crosstabulation IgM and IgG serologies in pregnant women who had a single test performed at our medical unit.

		IgG					
		Negative			Positive		
		*n*	%	95% CI	*n*	%	95% CI
IgM	negative	2322	9.69	9.32–10.07	21,304	88.88	88.47–89.27
	borderline	15	0.06	0.04–0.10	137	0.57	0.48–0.68
	positive	5	0.02	0.01–0.05	187	0.78	0.68–0.90
Total		2342	9.77	9.40–10.15	21,628	90.23	89.85–90.6

## Data Availability

The dataset used and/or analyzed during the present study can be obtained from the corresponding author on request.

## References

[1] Périllaud-Dubois C., Hachicha-Maalej N., Lepers C., Letamendia E., Teissier N., Cousien A., Sibiude J., Deuffic-Burban S., Vauloup-Fellous C., Picone O. (2023). Cost-effectiveness of screening and valacyclovir-based treatment strategies for first-trimester cytomegalovirus primary infection in pregnant women in France. Ultrasound Obs. Gynecol..

[2] Sanchez-Durán M.A., Maiz N., Liutsko L., Bielsa-Pascual J., García-Sierra R., Zientalska A.M., Velasco I., Vazquez E., Gracia O., Ribas A. (2023). Universal screening programme for cytomegalovirus infection in the first trimester of pregnancy: Study protocol for an observational multicentre study in the area of Barcelona (CITEMB study). BMJ Open.

[3] Pontes K.F.M., Nardozza L.M.M., Peixoto A.B., Werner H., Tonni G., Granese R., Araujo Júnior E. (2024). Cytomegalovirus and Pregnancy: A Narrative Review. J. Clin. Med..

[4] Ssentongo P., Hehnly C., Birungi P., Roach M.A., Spady J., Fronterre C., Wang M., Murray-Kolb L.E., Al-Shaar L., Chinchilli V.M. (2021). Congenital Cytomegalovirus Infection Burden and Epidemiologic Risk Factors in Countries With Universal Screening: A Systematic Review and Meta-analysis. JAMA Netw. Open.

[5] Monteiro S., Gonçalves A., Torrão M.M., Costa V., Almeida A. (2023). Knowledge of cytomegalovirus and available prevention strategies in pregnancy: A cross-sectional study in Portugal. J. Matern. Fetal Neonatal Med..

[6] Chatzakis C., Ville Y., Makrydimas G., Dinas K., Zavlanos A., Sotiriadis A. (2020). Timing of primary maternal cytomegalovirus infection and rates of vertical transmission and fetal consequences. Am. J. Obstet. Gynecol..

[7] Buca D., Di Mascio D., Rizzo G., Giancotti A., D’Amico A., Leombroni M., Makatsarya A., Familiari A., Liberati M., Nappi L. (2021). Outcome of fetuses with congenital cytomegalovirus infection and normal ultrasound at diagnosis: Systematic review and meta-analysis. Ultrasound Obs. Gynecol..

[8] Rybak-Krzyszkowska M., Górecka J., Huras H., Massalska-Wolska M., Staśkiewicz M., Gach A., Kondracka A., Staniczek J., Górczewski W., Borowski D. (2023). Cytomegalovirus Infection in Pregnancy Prevention and Treatment Options: A Systematic Review and Meta-Analysis. Viruses.

[9] Revello M.G., Tibaldi C., Masuelli G., Frisina V., Sacchi A., Furione M., Arossa A., Spinillo A., Klersy C., Ceccarelli M. (2015). Prevention of primary cytomegalovirus infection in pregnancy. EBioMedicine.

[10] Korver A.M., de Vries J.J., de Jong J.W., Dekker F.W., Vossen A.C., Oudesluys-Murphy A.M. (2009). Awareness of congenital cytomegalovirus among doctors in the Netherlands. J. Clin. Virol..

[11] Goderis J., De Leenheer E., Smets K., Van Hoecke H., Keymeulen A., Dhooge I. (2014). Hearing loss and congenital CMV infection: A systematic review. Pediatrics.

[12] Avettand-Fenoel V., Magny J.F., Ville Y., Leruez-Ville M. (2013). Utilisation des tests virologiques pour le diagnostic, le pronostic et la surveillance des infections congénitales à cytomégalovirus [Virological tools for the diagnosis, the prognosis and the surveillance of congenital cytomegalovirus infections]. Arch. Pediatr..

[13] Salomè S., Corrado F.R., Mazzarelli L.L., Maruotti G.M., Capasso L., Blazquez-Gamero D., Raimondi F. (2023). Congenital cytomegalovirus infection: The state of the art and future perspectives. Front. Pediatr..

[14] Razonable R.R., Humar A. (2019). Cytomegalovirus in solid organ transplant recipients-Guidelines of the American Society of Transplantation Infectious Diseases Community of Practice. Clin. Transplant..

[15] Davis N.L., King C.C., Kourtis A.P. (2017). Cytomegalovirus infection in pregnancy. Birth Defects Res..

[16] Alford C.A., Stagno S., Pass R.F., Britt W.J. (1990). Congenital and perinatal cytomegalovirus infections. Rev. Infect. Dis..

[17] Zuhair M., Smit G.S.A., Wallis G., Jabbar F., Smith C., Devleesschauwer B., Griffiths P. (2019). Estimation of the worldwide Seroprevalence of cytomegalovirus: A systematic review and meta-analysis. Rev. Med. Virol..

[18] Leruez-Ville M., Foulon I., Pass R., Ville Y. (2020). Cytomegalovirus infection during pregnancy: State of the science. Am. J. Obstet. Gynecol..

[19] Choodinatha H.K., Jeon M.R., Choi B.Y., Lee K.N., Kim H.J., Park J.Y. (2023). Cytomegalovirus infection during pregnancy. Obstet. Gynecol. Sci..

[20] Venturini C., Breuer J. (2025). Cytomegalovirus Genetic Diversity and Evolution: Insights into Genotypes and Their Role in Viral Pathogenesis. Pathogens.

[21] Xie M., Tripathi T., Holmes N.E., Hui L. (2023). Serological screening for cytomegalovirus during pregnancy: A systematic review of clinical practice guidelines and consensus statements. Prenat. Diagn..

[22] Kenneson A., Cannon M.J. (2007). Review and meta-analysis of the epidemiology of congenital cytomegalovirus (CMV) infection. Rev. Med. Virol..

[23] Harmon C.M., Cooling L.L. (2017). Current strategies and future directions for the prevention of transfusion-transmitted cytomegalovirus. Int. J. Clin. Transfus. Med..

[24] Balegamire S.J., McClymont E., Croteau A., Dodin P., Gantt S., Besharati A.A., Renaud C., Mâsse B., Boucoiran I. (2022). Prevalence, incidence, and risk factors associated with cytomegalovirus infection in healthcare and childcare worker: A systematic review and meta-analysis. Syst. Rev..

[25] Adler S.P. (2011). Screening for Cytomegalovirus during Pregnancy. Infect. Dis. Obstet. Gynecol..

[26] Khalil A., Heath P.T., Jones C.E., Soe A., Ville Y.G. (2025). Royal College of Obstetricians Gynaecologists Congenital Cytomegalovirus Infection: Update on Screening Diagnosis Treatment: Scientific Impact Paper No 56. BJOG.

[27] Permar S.R., Schleiss M.R., Plotkin S.A. (2025). A vaccine against cytomegalovirus: How close are we?. J. Clin. Investig..

[28] Zammarchi L., Tomasoni L.R., Liuzzi G., Simonazzi G., Dionisi C., Mazzarelli L.L., Seidenari A., Maruotti G.M., Ornaghi S., Castelli F. (2023). MEGAL-ITALIWorking Group Treatment with valacyclovir during pregnancy for prevention of congenital cytomegalovirus infection: A real-life multicenter Italian observational study. Am. Jobstet. Gynecol. MFM.

[29] Faas B.H.W., Astuti G., Melchers W.J.G., Reuss A., Gilissen C., Macville M.V.E., Ghesquiere S.A.I., Houben L.M.H., Srebniak M.I., Geeven G. (2024). Early detection of active Human CytomegaloVirus (hCMV) infection in pregnant women using data generated for noninvasive fetal aneuploidy testing. EBioMedicine.

[30] Maltezou P.G., Kourlaba G., Kourkouni Ε., Luck S., Blázquez-Gamero D., Ville Y., Lilleri D., Dimopoulou D., Karalexi M., Papaevangelou V. (2020). Maternal type of CMV infection and sequelae in infants with congenital CMV: Systematic review and meta-analysis. J. Clin. Virol..

[31] Hui L., Shand A. (2021). Is it time to adopt routine cytomegalovirus screening in pregnancy? No. Am. J. Obstet. Gynecol. MFM.

[32] Enders G., Daiminger A., Bäder U., Exler S., Enders M. (2011). Intrauterine transmission and clinical outcome of 248 pregnancies with primary cytomegalovirus infection in relation to gestational age. J. Clin. Virol..

[33] Department of Health (2020). Clinical Practice Guidelines: Pregnancy Care.

[34] Royal Australian and New Zealand College of Obstetricians and Gynaecologists (2019). Prevention of Congenital Cytomegalovirus (CMV) Infection [Internet].

[35] Gyselaers W., Jonckheer P., Ahmadzai N., Ansari M.T., Carville S., Dworzynski K., Gaudet L., Glen J., Jones K., Miller P. (2015). What Are the Recommended Clinical Assessment and Screening Tests during Pregnancy? Good Clinical Practice (GCP).

[36] Boucoiran I., Yudin M., Poliquin V., Caddy S., Gantt S., Castillo E. (2021). Guideline no. 420: Cytomegalovirus infection in pregnancy. J. Obstet. Gynaecol. Can..

[37] (2015). Congenital Cytomegalovirus Clinical Practice Guideline [Internet]; King Edward Memorial Hospital Obstetrics & Gynaecology: Subiaco, Australia. https://onlinelibrary.wiley.com/doi/pdf/10.1111/jpc.15946.

[38] Siennicka J., Dunal-Szcepaniak M., Trzcińska A., Godzik P., Rosińska M. (2017). High Seroprevalence of CMV Among Women of Childbearing Age Implicates High Burden of Congenital Cytomegalovirus Infection in Poland. Pol. J. Microbiol..

[39] Radoi C.L., Zlatian O., Balasoiu M., Dragomir T.L., Sorop M.I., Bagiu I.C., Boeriu E., Susan M., Sorop B., Oprisoni L.A. (2024). Seroprevalence of Anti-Cytomegalovirus Antibodies in Pregnant Women from South-West Romania. Microorganisms.

[40] Gorun F., Motoi S., Malita D., Navolan D.B., Nemescu D., Olariu T.R., Craina M., Vilibic-Cavlek T., Ciohat I., Boda D. (2020). Cytomegalovirus seroprevalence in pregnant women in the western region of Romania: A large-scale study. Exp. Ther. Med..

[41] Briciu V., Ionica A.M., Flonta M., Almas A., Muntean M., Topan A., Horvat M., Ungureanu L., Lupse M. (2023). Toxoplasmosis Screening during Pregnancy in a Romanian Infectious Diseases Tertiary Center: Results of a 15 Years Follow-Up Program. Microorganisms.

[42] Protocol Regarding the Methodology of Prenatal and Postnatal Consultations Documented in the Pregnant Woman’s Card. https://legislatie.just.ro/Public/DetaliiDocumentAfis/246408.

[43] Mocanu A.G., Gorun F., Ciohat I., Navolan D., Malita D., Vilibic-Cavlek T., Dahma G., Neamtu R., Popescu D., Cioca A. (2021). Simultaneous Seroprevalence to *Toxoplasma gondii*, Cytomegalovirus and Rubella Virus in Childbearing Women from Western Romania. Medicina.

[44] Antona D., Lepoutre A., Fonteneau L., Baudon C., Halftermeyer-Zhou F., LEStrat Y., Lévy-Bruhl D. (2017). Seroprevalence of cytomegalovirus infection in France in 2010. Epidemiol. Infect..

[45] Barlinn R., Dudman S.G., Rollag H., Trogstad L., Lindstrøm J.C., Magnus P. (2022). Maternal cytomegalovirus infection and delayed language development in children at 3 years of age-a nested case-control study in a large population-based pregnancy cohort. PLoS ONE.

[46] D’Antonio F., Marinceu D., Prasad S., Khalil A. (2023). Effectiveness and safety of prenatal valacyclovir for congenital cytomegalovirus infection: Systematic review and meta-analysis. Ultrasound Obs. Gynecol..

[47] Shahar-Nissan K., Pardo J., Peled O., Krause I., Bilavsky E., Wiznitzer A., Hadar E., Amir J. (2020). Valaciclovir to prevent vertical transmission of cytomegalovirus after maternal primary infection during pregnancy: A randomised, double-blind, placebo-controlled trial. Lancet.

[48] Razonable R.R., Inoue N., Pinninti S.G., Boppana S.B., Lazzarotto T., Gabrielli L., Simonazzi Pellett P.E., Schmid D.S. (2020). Clinical Diagnostic Testing for Human Cytomegalovirus Infections. J. Infect. Dis..

[49] Barber V., Calvert A., Vandrevala T., Star C., Khalil A., Griffiths P., Heath P.T., Jones C.E. (2020). Prevention of Acquisition of Cytomegalovirus Infection in Pregnancy Through Hygiene-based Behavioral Interventions: A Systematic Review and Gap Analysis. Pediatr. Infect. Dis, J..

[50] Rodríguez-Muñoz M.F., Martín-Martín C., Kovacheva K., Olivares M.E., Izquierdo N., Pérez-Romero P., García-Ríos E. (2024). Hygiene-based measures for the prevention of cytomegalovirus infection in pregnant women: A systematic review. BMC Pregnancy Childbirth.

[51] Calvert A., Vandrevala T., Parsons R., Barber V., Book A., Book G., Carrington D., Greening V., Griffiths P., Hake D. (2021). Changing knowledge, attitudes and behaviours towards cytomegalovirus in pregnancy through film-based antenatal education: A feasibility randomised controlled trial of a digital educational intervention. BMC Pregnancy Childbirth.

[52] Sankey MATIC. https://sankeymatic.com.

[53] Mocanu A.G., Stoian D.L., Daescu A.C., Motofelea A.C., Ciohat I.M., Navolan D.B., Vilibic-Cavlek T., Bogdanic M., Nemescu D., Tomescu L. (2024). The Impact of Latent Cytomegalovirus Infection on Spontaneous Abortion History and Pregnancy Outcomes in Romanian Pregnant Women. Microorganisms.

[54] Mussi-Pinhata M.M., Yamamoto A.Y., Aragon D.C., Duarte G., Fowler K.B., Boppana S., Britt W.J. (2018). Seroconversion for Cytomegalovirus Infection During Pregnancy and Fetal Infection in a Highly Seropositive Population: “The BraCHS Study”. J. Infect. Dis..

[55] Izquierdo G., Sandoval A., Abarzúa F., Yamamoto M., Rodríguez J.G., Silva M., Torres J.P., Aravena M., Fuentes D., Reyes A. (2021). Recomendaciones para el diagnóstico y manejo de la infección por citomegalovirus en la mujer embarazada y el recién nacido. Rev. Chil. Infectol..

[56] Hadjiiona A., Michaelides I., Kummer P., Kappelmeyer M., Koeninger A., Reuschel E. (2025). Frequency of CMV testing during pregnancy-a retrospective study. Arch. Gynecol. Obstet..

[57] Enders G., Daiminger A., Lindemann L., Knotek F., Bäder U., Exler S., Enders M. (2012). Cytomegalovirus (CMV) seroprevalence in pregnant women, bone marrow donors and adolescents in Germany, 1996–2010. Med. Microbiol. Immunol..

[58] Greye H., Wex T., Taneva E., Redlich A., Costa S.-D., Rissmann A. (2023). Cytomegalovirus seronegativity rate in pregnant women and primary cytomegalovirus infection during pregnancy in rural Germany. BMC Pregnancy Childbirth.

[59] De Vries J.J., van Zwet E.W., Dekker F.W., Kroes A.C., Verkerk P.H., Vossen A.C. (2013). The apparent paradox of maternal seropositivity as a risk factor for congenital cytomegalovirus infection: A population-based prediction model. Rev. Med. Virol..

[60] Billette de Villemeur A., Hoen B., Billaud E., Deruelle P., Goueslard K., Halley des Fontaines V., Minodier P., Parent B., Pozzetto B., Revest M. (2024). Current evidence gaps to support systematic cytomegalovirus screening in pregnancy. eClinicalMedicine.

[61] Bartnik P., Bender A., Kacperczyk-Bartnik J., Ciebiera M., Urban A., Sienko A., Bilir E., Romejko-Wolniewicz E., Sieńko J. (2024). Awareness of Pregnant Patients about Congenital Cytomegalovirus Infection—A Semi-Systematic Review. J. Clin. Med..

[62] Greye H., Henning S., Freese K., Köhn A., Lux A., Radusch A., Redlich A., Schleef D., Seeger S., Thäle V. (2022). Cross-sectional study to assess awareness of cytomegalovirus infection among pregnant women in Germany. BMC Pregnancy Childbirth.

[63] Boppana S.B., Rivera L.B., Fowler K.B., Mach M., Britt W.J. (2001). Intrauterine transmission of cytomegalovirus to infants of women with preconceptional immunity. N. Engl. J. Med..

[64] Leruez-Ville M., Chatzakis C., Lilleri D., Blazquez-Gamero D., Alarcon A., Bourgon N., Foulon I., Fourgeaud J., Gonce A., Jones C.E. (2024). Consensus recommendation for prenatal, neonatal and postnatal management of congenital cytomegalovirus infection from the European congenital infection initiative (ECCI). Lancet Reg. Health Eur..

[65] Linthorst J., Baksi M.M.M., Welkers M.R.A., Sistermans E.A. (2023). The cell-free DNA virome of 108,349 Dutch pregnant women. Prenat. Diagn..

